# A systematic review and meta-analysis of the efficacy and safety of western medicine routine treatment combined with Chinese herbal medicine in the treatment of COVID-19

**DOI:** 10.1097/MD.0000000000021616

**Published:** 2020-08-07

**Authors:** Xuemei Wang, Ping Xie, Guojuan Sun, Zhumei Deng, Min Zhao, Shuting Bao, Yunxia Zhou

**Affiliations:** a Department of Gynecology, Hospital of Chengdu University of Traditional Chinese Medicine, College of Clinical Medicine, Chengdu University of Traditional Chinese Medicine; b Department of Gynecology, Hospital of Chengdu University of Traditional Chinese Medicine, Chengdu, Sichuan Province, P. R. China.

**Keywords:** Chinese herbal medicine, coronavirus disease 2019, COVID-19, meta-analysis, systematic review, TRADITIONAL Chinese medicine, western medicine

## Abstract

**Background:**

COVID-19 is a global public health emergency. At present, there is no highly effective medicine for the prevention and treatment of 2019-nCoV. Western medicine for COVID-19 is mainly based on symptomatic support therapy. Chinese herbal medicine has been used to prevent infectious diseases for thousands of years in China. Western medicine routine treatment combined with Chinese herbal medicine is an alternative clinical option but lacks evidence-based medical evidence. The systematic review protocol aims to formulate a research plan that can evaluate the efficacy and safety of western medicine routine treatment combined with Chinese herbal medicine for COVID-19.

**Methods:**

We will search the following eight databases: Cochrane Library, PubMed, Embase, Medline, CNKI, Wanfang, VIP, and CBM. The search time is up to the end of July 2020. Two authors will independently complete literature screening, data extraction, and risk of bias assessment. In case of disagreement, the third author will assist in the judgment. The primary outcome will be the clinical cure rate. The secondary outcome will be accounting symptoms, fever time, time of virus nucleic acid turning negative, check the condition by drawing blood, pneumonia absorption rate, patient hospitalization time, severe conversion rate and case fatality rate, adverse reactions, and adverse events. Revman 5.3 will be used for systematic reviews and meta-analysis. The report of the protocol will follow the PRISMA-P statement, and the report of the systematic review and meta-analysis will follow the PRISMA statement.

**Results:**

We will provide evidence-based medical evidence of the efficacy and safety of western medicine routine treatment combined with Chinese herbal medicine for COVID-19. The findings will be published in peer-reviewed journals.

**Registration details:**

CRD42020190106.

## 1 Introduction

Corona Virus Disease 2019 (COVID-19) was the first reported in Wuhan, China, on December 8, 2019. Since then, the epidemic has rapidly swept the world.^[1]^ The number of diagnoses, deaths, and new cases in the world is increasing, which has become a severe public health emergency endangering the planet. The novel coronavirus mainly spreads through respiratory droplets and contact, with the characteristics of the long incubation period, fast transmission speed, and susceptibility to all ages, which poses great challenges to the global epidemic prevention system.^[2]^ Because the novel coronavirus epidemic is highly infectious, epidemic, and harmful, it greatly hinders the normal progress of human production, life, business, and social order. It is one of the plagues that seriously endanger people's health, public safety, and social development.^[3]^

Coronavirus is a kind of enveloped RNA virus, which is widely distributed in human, mammal, and bird hosts. It mainly causes respiratory diseases, but also gastrointestinal, liver, and nervous system diseases. Coronavirus can cause severe epidemic diseases such as cold, Severe Acute Respiratory Syndrome (SARS), and Middle East Respiratory Syndrome (MERS) after infecting the human host.^[4]^ SARS-CoV-2 belongs to the genus β-coronavirus. Genomic studies have found that the sequence of SARS-CoV-2 is 40% to 70% similar to the sequence of SARS-CoV and MERS-CoV.^[5]^ The common symptoms of humans infected with novel coronavirus are fever, sore throat, cough, fatigue, shortness of breath, dyspnea, etc in severe cases, diffuse pneumonia, acute respiratory syndrome, multiple organ failure, shock, and even death may occur.^[6]^

At present, there are no highly effective drugs for the prevention and treatment of novel coronaviruses. The clinical treatment of COVID-19 in Western medicine is mainly based on the experience of the previous treatment of SARS, HIV, MERS, and other viruses, mostly by antiviral, anti-infection, antipyretic, oxygen inhalation, nutrition, fluid replacement, and other non-specific treatment measures to prevent disease progression, reduce the conversion rate of mild to severe disease and mortality, and improve the success rate of disease treatment.^[7–9]^

Chinese herbal medicine has a history of preventing and controlling infectious diseases in China for thousands of years. During this period, traditional Chinese medicine has waged a long-term struggle against infectious diseases such as plague, cholera, and smallpox, and has accumulated valuable experience.^[10]^ In recent years, traditional Chinese medicine has also achieved outstanding results in combating SARS, AIDS, and H1N1 influenza.^[11]^ COVID-19 belongs to the category of plague in traditional Chinese medicine. The primary pathogenesis of the disease is dampness, and the disease location is mainly in the lung.^[12]^ The Chinese government actively promotes the full participation of traditional Chinese medicine in the prevention and control of the epidemic, enhances the curative effect with the combination of traditional Chinese and Western medicine, and gives full play to the role of traditional Chinese medicine in the prevention and control of the epidemic.^[13]^ During the epidemic, the clinical trial of Western medicine combined with Chinese herbal medicine treatment in COVID-19 is increasing, so it is necessary to carry out this work.

## 2 Objectives

This systematic review aims to explore the efficacy and safety of the combination of Western and Chinese medicine in the treatment of COVID-19, to provide evidence-based medical evidence for the prevention and treatment of this epidemic.

## 3 Methods

This work is a systematic review of published clinical studies. If necessary, meta-analysis will be possible. The data used in this systematic review will be all from published literature. Therefore, there is no need to provide ethical approval.

### 3.1 Registration and protocol

This work has been registered on the PROSPERO international registration platform, with the registration number of CRD42020190106.

The protocol of systematic review is written strictly under the relevant requirements in PRISMA-P (Preferred Reporting Items for Systematic Review and Meta-Analysis Protocols),^[14,15]^ and the reporting of the results of the systematic review will strictly follow the PRISMA statement.

### 3.2 Retrieve resources

#### 3.2.1 Database search and search strategy

We will use electronic computer retrieval to search English databases, including Cochrane Library, PubMed, Embase, Medline (via OvidSP), and Chinese databases, including CNKI, Wanfang, VIP, and CBM. The search time is from the database establishment to July 2020. At the end of July, we will complete the initial retrieval. Before completing the systematic review, we will retrieve the database again and update the retrieval results if necessary.

Each database will use subject words and free words to search. Table 1 lists the detailed search strategies and search words of the PubMed database. The search strategies of other databases will convert the logical operators and search fields accordingly. In Chinese database search, the English search terms in PubMed search strategy are translated into Chinese form.

**Table 1:**
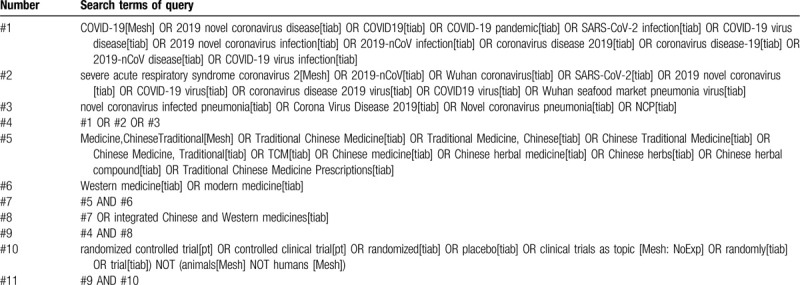
Search strategy for PubMed.

#### 3.2.2 Other search resources

We will continue to search the clinical trial registration platforms (Clinicaltrials.gov, WHO ICTRP, Chinese Clinical Trial Register, ISRCTN, UK National Research Register, UK Clinical Trial Gateway, and UMIN CTR) to obtain the clinical trial data under research or not published. We will search Baidu Scholar and Google Scholar to get the full text of the literature.

### 3.3 Inclusion and exclusion criteria

#### 3.3.1 Study design

We will include randomized controlled trials or Quasi-Experimental studies of western medicine routine treatment combined with Chinese herbal medicine for the treatment of COVID-19. Review literature, experience summary, theoretical discussion, animal experiments, case reports, observational studies, and retrospective studies will be excluded. The studies were published in English and Chinese only.

#### 3.3.2 Participants

Patients diagnosed with COVID-19 will be included. The diagnostic criteria refer to clinical diagnosis and treatment guidelines issued by the United States^[16]^ and China.^[17]^ The diagnostic details of COVID-19 include

1. epidemiological history: people who have been to the epidemic area or contacted with novel coronavirus infection within 14 days before the onset of illness;
2. clinical manifestations: patients with fever, respiratory symptoms, and novel coronavirus pneumonia imaging characteristics;
3. laboratory examination: real-time fluorescent RT-PCR detection of novel coronavirus nucleic acid positive.

Regardless of the patient's course of disease, severity of illness, country, skin color, age, work, and education level.

#### 3.3.3 Interventions

Regardless of the order of the experimental group and the control group in the original clinical study, all the clinical studies included in this systematic review, the intervention group's intervention measures will be defined as western medicine routine treatment combined with Chinese herbal medicine. The intervention measures of the control group will be defined as western medicine routine treatment. Western medicine routine treatment for COVID-19 includes anti-virus, anti-infection, antipyretics, oxygen inhalation, fluid replacement, circulation support, renal replacement, immunotherapy, etc. Chinese herbal medicine treatment includes Chinese medicine compound, Chinese patent medicine, Chinese medicine decoction, Chinese medicine injection, etc. We will exclude interventions such as acupuncture, moxibustion, acupoint application, Chinese medicine ironing, Chinese medicine foot bath, and other Chinese medicine therapy. Does not limit the duration and dosage of interventions?

#### 3.3.4 Outcomes

##### 3.3.4.1 Primary outcome

The primary outcome will be defined as clinical cure rate. The proportion of clinically cured patients will be used to measure the clinical cure rate.

##### 3.3.4.2 Secondary outcome

1. Accompanying symptoms.
2. After treatment, the change of the accompanying symptoms was measured by symptom score.
3. Fever time.
4. The time required for the patient's body temperature to return to normal after treatment, measured in days.
5. Time of virus nucleic acid turning negative.^[18]^
6. The time after the treatment of novel coronavirus nucleic acid changes from positive to negative.
7. Check the condition by drawing blood.^[19]^
8. The content of the blood sample test will include WBC, LYM, LYM%, CRP, PCT, etc. The proportion of patients whose blood sample indexes returns to normal after treatment accounts for the total number of patients.
9. Pneumonia absorption rate.^[20]^
10. After treatment, CT showed the rate of inflammation absorption in the patient's lungs.
11. Patient hospitalization time
12. Time from admission to discharge.
13. Severe conversion rate, case fatality rate.
14. After treatment, the proportion of patients’ condition aggravating, transforming into severe type, and the proportion of patients who died of invalid treatment.
15. Adverse reactions and adverse events.
16. Adverse reactions and events recorded in the included clinical trial.

### 3.4 Literature screening

**Figure 1:**
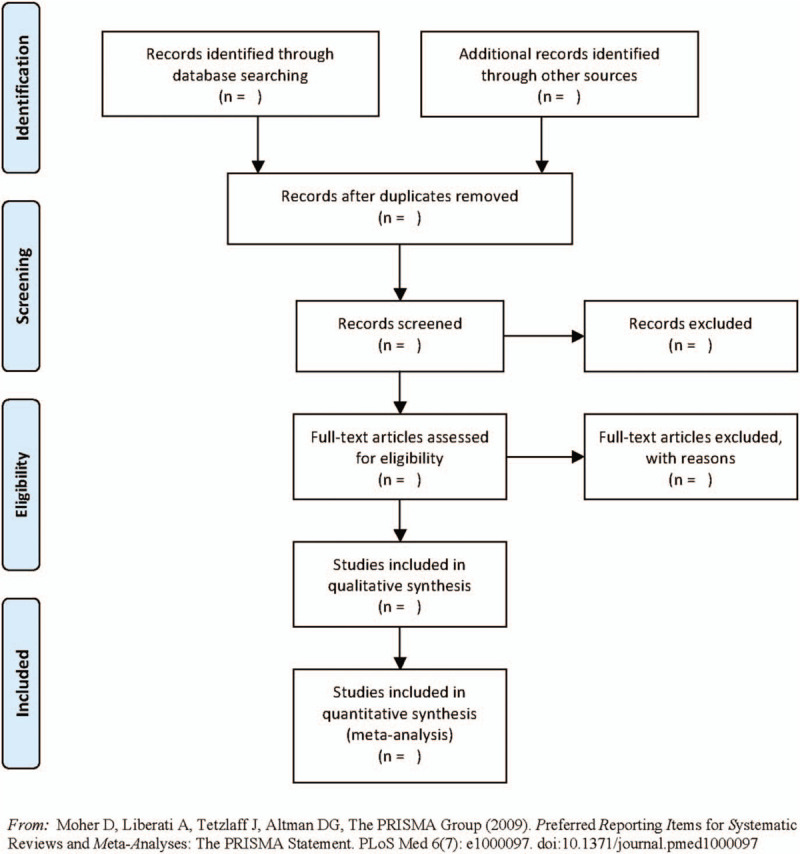
The PRISMA flow diagram of literature screening process.

EndNote X9 software will be used to import, group, deduplicate, add full text, and other management. After importing the bibliography into EndNote, we will first conduct repetitive screening of the literature by comparing the title, author, year, journal name, volume, page number, and other information, remove the duplicate bibliography. Two authors (MZ, ZD) trained in evidence-based medicine review the titles and abstracts of the bibliography one by one according to the inclusion and exclusion criteria, and move the bibliography that obviously does not meet the inclusion criteria into the exclusion folder. Add the full text of the bibliography that meets the requirements of the preliminary review. The two authors (MZ, ZD) will read the content of the study design in the full text of the literature one by one, move the literature that does not meet the requirements to the exclusion folder, and record the reasons for the exclusion in detail in Excel. In the process of screening, if there is any disagreement, it should be solved by discussion first. If the disagreement still exists, the author (XW) will assist in the judgment. The literature screening process is shown in Figure 1.

### 3.5 Data extraction

After discussion, all authors will decide the literature data extraction content and make a literature extraction table. Before formal literature extraction, 5 to 6 pieces of literature will be selected for data extraction tests to verify whether the literature extraction table's structure is complete, convenient, and reliable. Formal literature extraction will be conducted by two authors (YZ, SB) complete independently. In case of any difference, it will be discussed and resolved first. If the difference still exists, the author (XW) will assist in judgment. If the literature data records are incomplete, we will contact the author by email and try our best to complete the information. The structure of literature extraction table is as follows:

1. Basic information: literature number, literature title, first author, country and region, year of publication, corresponding author email, fund, full name of a journal, the language of publication, ethical approval, conflict of interest;
2. Study methods: diagnostic criteria, study design type, random method, scheme concealment, sample size, blinding method, follow-up time, research data integrity, statistical method;
3. Patients information: age, course, race, baseline level;
4. Intervention information: types of intervention measures, intervention time, intervention frequency;
5. Outcome information: primary/secondary outcome values, measurement methods, measurement time, adverse reactions, adverse events.

### 3.6 Risk of bias assessment

Refer to Cochrane Review Handbook 5.1.0 for the risk assessment of bias to evaluate the quality of the literature included in this study.^[21]^ Two authors (PX, GS) independently assess the quality of literature and jointly check the evaluation results. If the evaluation results are inconsistent, it shall be solved through discussion first. If it still cannot be resolved, it shall be judged by the third author (XW). The contents of the risk assessment of bias will include random methods, scheme concealment, blind participants, blind analysis of results, outcome data integrity, selective reporting, etc. The bias is divided into three levels: high risk, low risk, and unclear. The evaluation results will be entered in Review Manager 5.3 software to generate specific graphs. If the information about the risk of bias in the clinical trial is unclear, we will try to contact the author by email.

### 3.7 Data analysis and synthesis

According to the heterogeneity between studies, the method of data analysis and synthesis is determined. If the heterogeneity between the included studies is within the acceptable range, a meta-analysis of the study results will be conducted. If the heterogeneity between the included studies is significant, we will take a descriptive analysis of the study results. Chi-square test (χ^2^) and *I*^2^ will be used to analyze the heterogeneity between the clinical trials. If *P* > .1, *I*^2^ ≤ 50%, it indicates that the heterogeneity between the clinical trials is within the acceptable range, and a fixed-effect model will be used to analyze the data. If *P* ≤ .1, *I*^2^ > 50%, indicating that the heterogeneity between clinical trials is considerable, subgroup analysis will be needed to identify the source of heterogeneity, and the random effect model will be used to analyze the data. If *P* ≤ .1, *I*^2^ > 75%, it indicates that the heterogeneity between the clinical trials is too significant, and only descriptive analysis can be used. RevMan5.3 software will be used to synthesize the study data. The odds ratio (OR) and 95% confidence interval (CI) are used to describe the effect size of the binary data. Mean difference (MD) or standardized mean difference (SMD) and 95% confidence interval (CI) are used to describe the effect size of continuous data. The *Z* test judges the effect size, and it has statistical significance when *P* ≤ .05. The data synthesis results will be presented in the form of forest plots.

### 3.8 Subgroup analysis

If there is a certain degree of heterogeneity between included clinical trials, subgroup analysis can be used to determine the source of the heterogeneity. The subgroup analysis will be conducted according to the different types of western medicine routine treatment, the severity of the disease, different dosage, treatment courses, different outcome measurement time points, and different follow-up time points.

### 3.9 Sensitivity analysis

We will use sensitivity analysis to test the stability of the results of the meta-analysis study. The method is to exclude one study at a time, and then conduct a meta-analysis of the remaining studies. If the results of meta-analysis before and after elimination are quite different, the study results are not stable, and the study conclusions should be carefully analyzed.

### 3.10 Publication bias

If the number of clinical trials finally included is ≥10, the funnel plot will be made by Review Manager 5.3 software to detect publication bias. Egger shall be used to check the funnel plots’ symmetry.

## 4 Discussion

The outbreak of COVID-19 worldwide is a significant disaster that threatens human health.^[22]^ Although the etiology of COVID-19 has been clarified, the pathogenesis is still unclear, and there is currently no treatment for the cause.^[23]^ The SARS-CoV-2 pathogens are the same all over the world, and the pathogenic mechanism is the same, so the treatment plan is the same.^[24]^ However, due to differences in ethnicity, health habits of people in different countries, and the differences in medical treatment levels, the treatment options are varied. Western medicine treatment of COVID-19 plays a considerable role in the prevention and control of the world's epidemic situation. Still, there are also shortcomings: non-specific antiviral drugs, side effects of hormone therapy, excessive use of antibiotics, and other problems.^[25]^ Therefore, the Chinese government encourages doctors to fully consider the diagnosis and treatment of Western medicine and add traditional Chinese herbal medicine, to give full play to the advantages of western medicine routine treatment combined with Chinese herbal medicine for COVID-19.^[13]^

More and more clinical studies have shown that western medicine routine treatment combined with Chinese herbal medicine for COVID-19 has better efficacy than Western medicine alone.^[26,27]^ These include improving clinical recovery rate, relieving symptoms, reducing fever time, and improving immune function, promoting inflammation absorption, shortening hospitalization time, etc.^[28,29]^ However, due to the small sample size in existing studies, it isn’t easy to draw reliable conclusions on the efficacy and safety of a certain therapy. By conducting systematic reviews and meta-analysis of multiple controlled studies of the two therapies, the statistical power is increased, to obtain a reliable conclusion from the existing clinical trials of COVID-19, and provide evidence-based medical evidence for the treatment of COVID-19.

There are some limitations to this systematic review. First, the outbreak of COVID-19 is sudden. It is impossible to formulate and implement a large-sample randomized controlled trial in a short period, because the sample size is not large enough and the quality of clinical trials may not be high enough, which affects the quality of evidence to a certain extent; secondly, the different types, dosage forms, and dosages of traditional Chinese herbal medicine may cause clinical heterogeneity; thirdly, due to the unfamiliarity with other languages and database access rights in other languages, this review will only include in Chinese and English studies literature, which may lead to selective bias. Although there are some limitations, the team members still carry out this review to provide some references and suggestions for clinical decision-making and further clinical research.

## Acknowledgments

Thanks to the National Natural Science Foundation of China for funding this work (project No. 81674017).

## Author contributions

**Conceptualization:** Xuemei Wang, Ping Xie, Guojuan Sun, Shuting Bao.

**Data curation:** Xuemei Wang, Ping Xie, Yunxia Zhou, Shuting Bao.

**Formal analysis:** Xuemei Wang, Guojuan Sun, Min Zhao, Zhumei Deng.

**Funding acquisition:** Ping Xie, Guojuan Sun.

**Investigation:** Xuemei Wang, Guojuan Sun, Min Zhao, Yunxia Zhou.

**Methodology:** Xuemei Wang, Ping Xie, Guojuan Sun, Zhumei Deng.

**Project administration:** Xuemei Wang, Ping Xie, Guojuan Sun.

**Resources:** Xuemei Wang, Min Zhao, Zhumei Deng, Min Zhao, Zhumei Deng.

**Software:** Xuemei Wang, Guojuan Sun, Min Zhao, Yunxia Zhou.

**Supervision:** Xuemei Wang, Ping Xie, Guojuan Sun.

**Validation:** Xuemei Wang, Ping Xie, Guojuan Sun.

**Visualization:** Xuemei Wang, Ping Xie, Guojuan Sun.

**Writing – original draft:** Xuemei Wang.

**Writing – review & editing:** Ping Xie, Guojuan Sun.
